# Renal Cell Carcinoma: Prognosis in the Era of Targeted Therapy

**DOI:** 10.3390/curroncol32090515

**Published:** 2025-09-16

**Authors:** Kathrin Halfter, Michael Staehler, Dieter Hölzel, Alexander Crispin, Anne Schlesinger-Raab

**Affiliations:** 1 Institute of Medical Information Processing, Biometry and Epidemiology (IBE), Faculty of Medicine, LMU Munich, Marchioninistraße 15, 81377 Munich, Germany; 2 Department of Urology, LMU University Hospital, LMU Munich, Marchioninistraße 15, 81377 Munich, Germany

**Keywords:** renal cell carcinoma, kidney cancer, cancer epidemiology, targeted therapy, immunotherapy, time trends

## Abstract

**Background**: Significant changes in renal cell carcinoma (RCC) drug treatment and improved access to abdominal imaging have recently been implemented. The impact of these changes on patient characteristics and prognosis remains to be quantified. **Methods**: A population-based cohort of 210,418 RCC cases from the Centre for Cancer Registry Data (ZfKD) diagnosed in Germany between 2000 and 2019 was analyzed in this observational study. Three time periods of diagnosis were defined, the first (2000–2005) functioning as a control. The remaining were defined according to the introduction of tyrosine kinase targeting drugs (2006–2014) and checkpoint inhibitor drugs (2015–2019). Five-year relative survival (RS) trends for each risk group and metastatic RCC (mRCC) were determined using Poisson regression models. **Results**: Age at diagnosis and the proportion of low-risk disease increased, while the proportion of mRCC decreased (*p* < 0.0001). RS improved slightly between the first and last period in low (5-year RS 98.7% vs. 100.9%), intermediate (89.2% vs. 91.9%), and high-risk (76.6% vs. 80.3%), as well as mRCC (28.3% vs. 29.1%). The overall change in prognosis was significant in low (*p* = 0.0233) and high-risk groups (*p* = 0.0002), but not in intermediate-risk and mRCC groups. In a multivariate analysis, high-risk ccRCC patients appear to profit from drug treatment advances. **Conclusions**: Earlier detection has improved prognosis for the majority of RCC patients. Further efforts should be aimed at diagnosing more mRCC patients earlier, when surgical tumor removal remains feasible.

## Simple Summary

Incidental early detection plays an important role in renal carcinoma. A large cohort study of kidney cancer patients from Germany shows a trend towards smaller stages and fewer metastatic findings. This trend alone leads to an improved prognosis. Innovative systemic treatments show initial improvements in high-risk clear cell carcinomas, but not in metastatic patients.

## 1. Introduction

The availability of and access to abdominal imaging in western countries is largely attributed to an increasing incidence of small renal masses (SRM). These are in large part incidental findings (≈60%) and make up the majority of renal cell carcinoma (RCC) diagnoses [1,2]. SRM patients have an excellent prognosis with a five-year survival near 90% [3,4,5,6]. However, the prognosis for locally advanced or metastatic RCC (mRCC) is significantly lower. The reported 5-year relative survival (RS) for mRCC in Germany is between 19 and 21% [3,7]. It is therefore noteworthy that innovative treatment approaches have become available in the last two decades, such as drugs targeting the VEGF receptor (e.g., bevacizumab; European Medicines Agency (EMA) approval 2005 and U.S. Food and Drug Administration (FDA) approval 2009), the tyrosine kinase pathway (e.g., sunitinib or sorafenib; EMA and FDA approval 2006), and the mTOR pathway (e.g., temsirolimus; EMA and FDA approval 2007). Checkpoint inhibitors such as nivolumab (EMA and FDA approval 2015) or pembrolizumab (FDA approval 2014 and EMA approval 2015) replaced previous immunotherapy using interferon alpha [8,9]. The approvals for these drugs are mainly based on studies focusing on distinct subgroups such as advanced clear cell carcinoma (ccRCC). The results from the KEYNOTE-426/564 and Javelin Renal studies showed that newer drugs such as axitinib, pembrolizumab, cabozantinib, nivolumab, and ipilimumab have proven more effective in progression-free survival compared to tyrosine kinase inhibitors (TKI) such as sunitinib [10,11,12]. As promising as these studies appear, the gains are mainly based on improvements in progression-free survival, with only the KEYNOTE-564 reporting significant improvements in overall survival (OS) after immunotherapy versus placebo in ccRCC after nephrectomy [13]. Other checkpoint inhibitor trials (Immotion010/151, Checkmate 914, PROSPER, JAVELIN 101) reported significant results regarding disease-/recurrence-free survival or non-significant OS results [11,14,15,16]. The impact of these changes, both in drug treatment and in the increase in incidental diagnosis, remains to be quantified. In addition, real-world data that include less frequent histological subgroups remain scarce. Since RCC continues to rank as one of the 10 most frequently diagnosed malignancies, it is important to determine what treatment options are effective [17]. Especially considering the costs for the healthcare system associated with target treatment options, the effectiveness should be assessed in a population. Therefore, our aim was to analyze expected changes in demographics and prognosis in a large-scale cohort of RCC patients.

## 2. Patients and Methods

Patient-level data were retrieved from the German Centre for Cancer Registry Data (ZfKD) [17]. Patients aged 18 or older diagnosed between 2000 and 2019 with renal cell carcinoma were included in this analysis. Cases with death certificate only (DCO), benign, pediatric, or neuroendocrine tumors (NET), and nephroblastoma were excluded (Supplementary Figure S1). Histological classification was carried out according to the 2022 Edition of the WHO Classification of Tumours of the Urinary System and Male Genital Organs and the TNM—Classification version 8 [18,19]. The total time period was divided according to the approval and introduction of tyrosine kinase targeting drugs (2006–2014) and checkpoint inhibitor drugs (2015–2019). The first time period (2000–2005) was defined as the control period. These time intervals served as a surrogate parameter for treatment interventions due to the only recently available nationwide clinical cancer documentation (2020).

Patients with localized disease were grouped into University of California Los Angeles integrated staging system (UISS)-based risk categories according to clinical stage (TNM), grade, and histology: (1) Low, T1N0/NXM0G1-2; (2) intermediate, T1N0/NXM0G3-4, T2N0/NXM0G1-4, T3N0/NXM0G1, TXN0/NXM0G1-4; and (3) high, T3N0/NXM0G2-4, T4N0/NXM0G1-4, sarcomatoid histology [20]. mRCC (TN+M0G1-4, TNM+G1-4) was added as a fourth category. Documentation bias through varying data availability from individual cancer registries was considered throughout, and missing values are reported. This study follows the recommendations of the STROBE checklist for observational studies.

Data from all 16 cancer registries in Germany (one per federal state) were deemed appropriate and retrieved from the central administrative body, the German Centre for Cancer Registry Data (ZfKD) [17]. Information on the database, variables, and methods used for data collection and validity can be found on the center’s webpage (https://www.krebsdaten.de/Krebs/EN/Content/Methods/methods_node.html, accessed on 6 November 2020). Briefly, the Federal Cancer Registry Data Act legally requires full documentation of epidemiological data starting in 2009 (patient demographic, diagnosis, histological, and follow-up data). These data are collected by each federal state and sent to the ZfKD, where it is tested for plausibility and completeness. Access to this nationwide epidemiological dataset was granted following a successful submission and review of the project outline. Approval from the institutional ethics committee was granted (Project No. 24-0160 KB). No randomization, blinding, or sample size estimation was conducted since the analysis is based on retrospective population-level data. This study was registered under the public domain (https://edoc.rki.de/handle/176904/11012).

### 2.1. Outcome Measures

The main outcome was 5-year cumulative relative survival (RS). Significant 5-year RS trends were assessed in a Poisson model of relative excess risk of death (RER) adjusted for years of follow-up per UISS-risk category. The secondary outcome was cumulative OS and ccRCC/non-ccRCC subgroups.

### 2.2. Statistical Analyses

Survival was determined as the time from diagnosis until death from any cause. Cases without survival documentation were excluded. Follow-up was truncated after 10 years or censored at the end of the observation period (19 December 2019), whichever came first. RS was calculated as the ratio of OS and expected survival according to the cohort approach. Expected survival was determined using the Ederer II method and based on age- and sex-matched life tables of the German population [21]. Cumulative survival was obtained from a generated life table, and the 5-year survival is calculated by multiplying the prior survival rates for the 0 to 4 yearly intervals. Point estimates and 95% confidence intervals (CI) are reported.

Univariate and multiple Poisson regression was used to model trends in OS and RS [22]. The logarithm of the person-years at risk was included as an offset. Multiple Poisson regressions analyzing the effect of period of diagnosis were adjusted for follow-up interval, age, and sex.

A post hoc landmark (surviving at least one year) model selecting only cases of ccRCC, age at diagnosis less than 75, with documented surgery was performed as a sensitivity analysis to account for unknown comorbidities. First-year post-diagnosis mortality was analyzed using logistic regression. Pearson’s chi-square or Cochran–Armitage test for trend was used to compare categorical variables, and ANOVA for numerical variables. Respective effect sizes (Cramer’s *V* and Cohen’s *f*) are reported. Percentages for individual subgroups consider available data only, and missing values are given in relation to the underlying cohort or subgroup for the respective category. For all analyses, a two-sided *p*-value of 0.05 or less was considered statistically significant. The Statistical Analysis System SAS version 9.4 (SAS Institute, Cary, NC, USA) was used for data handling and analysis.

## 3. Results

### 3.1. Overall Demographic and Disease Characteristics

In this study, 210,418 patients were selected for demographic analyses (Supplementary Figure S1). As shown in Table 1, 63% of patients are male. Median age at diagnosis was 67 years (IQ-range 58–74) for men and 70 years (IQ-range 61–77) for women (*p* < 0.0001). Full UISS staging information was available in *n* = 125,659 (59.7%) cases. More than half of these patients were diagnosed with UISS low-risk disease (*n* = 66,369, 52.8%). UISS intermediate-risk was present in 12.9% (*n* = 16,206), high-risk in 13.9% (*n* = 17,516), and mRCC in 20.3% (*n* = 25,568) of patients. Low-risk disease was more common in women (55.0% vs. 51.6%), while distant metastasis was more frequently seen in men (16.8% vs. 14.6%; *p* < 0.0001).

### 3.2. Trends in the Patient Population

The absolute patient numbers increased annually by an average of 3.5% (95%CI[1.2–5.8], excluding two registries without complete surveys). The median age increased in line with an increase in life expectancy from the first period (2000–2005) with 66 years (IQ-range 59–73) to 69 years (IQ-range 59–75) in 2006–2014 and to 68 years (IQ-range 59–76) in 2015–2019 (*p* < 0.0001). The proportion of male patients has slightly increased from 63.0% to 65.9% (*p* < 0.0001). There is a trend toward earlier, localized, and regional stages regardless of histology, age, or sex (all *p* < 0.0001). In ccRCC the proportion of mRCC patients decreased from 20.8% to 15.8%, and in non-ccRCC from 24.0% to 21.2%. However, the effect size for these trends is small (all effect sizes < 0.2).

A shift from non-ccRCC (76.1% in 2000–2005, 52.6% in 2006–2014, 43.3% in 2015–2019) to ccRCC (23.9% in 2000–2005, 47.4% in 2006–2014, 56.7% in 2015–2019) was seen over time, aligning with guideline updates (*p* < 0.0001, Cramer’s V = 0.2294; WHO Classification of Urinary and Male Genital Tumours: 3rd Edition 2004, 4th Edition 2016, and 5th Edition 2022). Surgery was consistently performed on 90.4% of patients. Further analysis of clinical treatment was limited due to suboptimal treatment data (see Supplementary Table S1).

### 3.3. Survival Trends

A total of 176,076 patients were analyzed over a median time of 8.54 years (95%CI[8.46–8.54]); 34.1% of patients died (*n* = 60,133). The median age at death was 74 years (IQ-range 66–81). This resulted in an overall mortality rate of 63.8 per 1000 person-years (95%CI[63.2–64.3]). Five-year OS was 71.4% (95%CI[71.2–71.7]), and RS was 81.3% (95%CI[81.1–81.6]).

Mortality was the highest during the first-year post-diagnosis, with 31.7% (*n* = 19 053) of deaths occurring during this time. In a multiple logistic model, first-year mortality was associated with mRCC (OR 5.185 (95%CI[4.878–5.511]), *p* < 0.0001), no documented surgery (OR 2.343 (95%CI[2.149–2.556]), *p* < 0.0001), non-ccRCC histology (OR 1.305 (95%CI[1.223–1.391]), *p* < 0.0001), and female sex (OR 1.105 (95%CI[1.039–1.176]), *p* = 0.0015). Year of diagnosis was also associated with first-year mortality (OR 1.090 (95%CI[1.083–1.097]), *p* < 0.0001).

**Figure 1:**
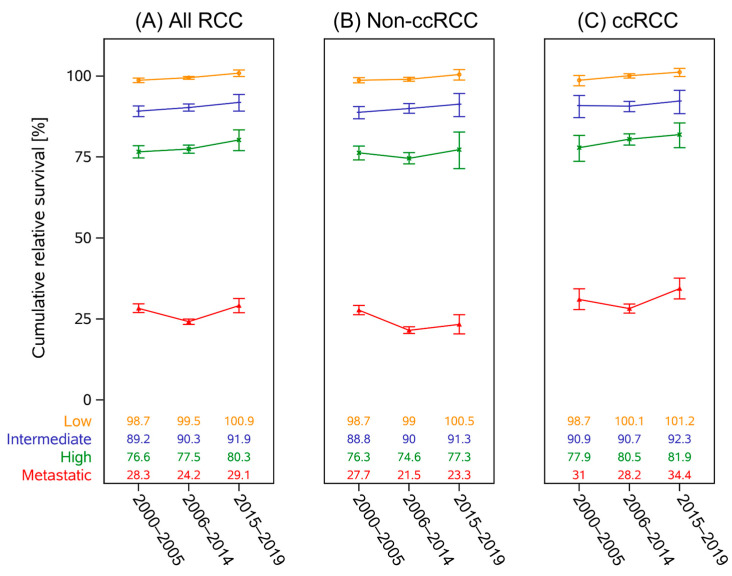
Unadjusted five-year cumulative RS point estimates and their 95% confidence intervals are shown according to risk category for each period of diagnosis. (**A**) All patients, (**B**) non-ccRCC, and (**C**) ccRCC histology subgroup. Median age at diagnosis for the three time periods in UISS-low patients was 65, 67, and 66 years. In the intermediate group, 65, 66, and 66 years, and 66, 69, and 69 years in the high-risk group. Patients with mRCC were a median of 65, 68, and 68 years old at diagnosis.

The change in point estimates of 5-year RS over the three time periods according to UISS-risk and RCC histology categories is shown in Figure 1. Significant differences comparing the three time periods were mainly found between the first and last periods. Increases in RS were significant for the low- (RER 0.395 (95%CI[0.210–0.972]); *p* = 0.0420) and high-risk groups (RER 0.772 (95%CI [0.670–0.891]); *p* = 0.0004). The change was not relevant in the intermediate risk group (RER 0.798 (95%CI [0.623–1.022]); *p* = 0.0744) and for mRCC (RER 1.051 (95%CI [0.999–1.104]); *p* = 0.0505).

In ccRCC, only the prognosis of the high-risk group showed a relevant improvement (RER 0.761 (95%CI[0.599–0.966]); *p* = 0.0248).

Prognosis for mRCC patients with non-ccRCC decreased between the first and second period (RER 1.204 (95%CI[1.148–1.262]); *p* < 0.0001) and between the first and last period (RER 1.244 (95%CI[1.169–1.323]); *p* < 0.0001).

**Figure 2:**
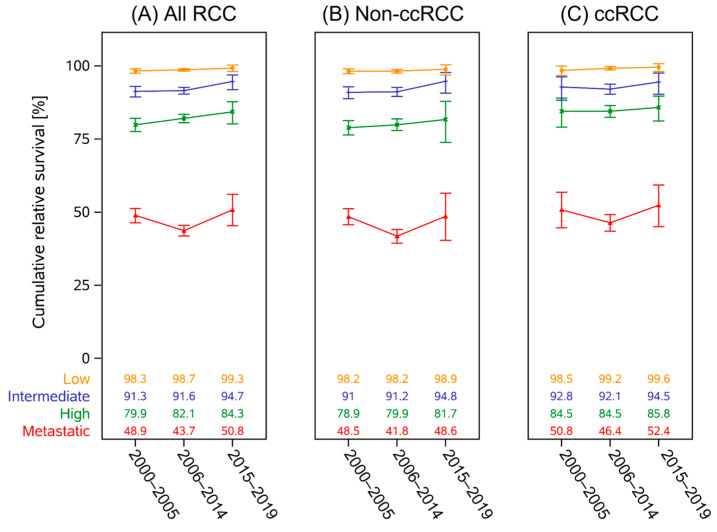
Results of the landmark model including only patients surviving the first year post-diagnosis, with documented surgery, and 75 years or younger at diagnosis. Five-year relative survival for all RCC (**A**), ccRCC (**B**), and non-ccRCC (**C**) according to UISS-based risk category.

These findings were in part upheld in a post hoc landmark analysis (*n* = 78 540; see Figure 2). Intermediate-risk (0.671 RER (95%CI[0.452–0.998]); *p* = 0.0488) and high-risk groups (RER 0.741 (95%CI[0.588–0.933]); *p* = 0.0108) showed significant improvement between the first and last periods. The decrease in RS for mRCC patients between the first and seconds period also remained significant (RER 1.140 (95%CI[1.052–1.236]); *p* = 0.0014). Within the RCC subcategories, this change in prognosis between the first and second period for non-ccRCC patients with distant metastasis continued to be significant (RER 1.179 (95%CI[1.073–1.295]); *p* = 0.0006). The results of the adjusted regression models are shown in Table 2.

For ccRCC, age was no longer significant in intermediate (RER 1.244 (95%CI[0.957–1.611]); *p* = 0.1039) and high-risk subgroups (RER 1.066 (95%CI[0.921–1.233]); *p* = 0.3920). In mRCC only age (RER 1.180 (95%CI[1.113–1.480]); *p* < 0.0001) remained a significant factor; the period of diagnosis was no longer relevant (2006–2014 RER 1.048 (95%CI [0.957–1.146]); *p* = 0.3108, 2015–2019 RER 0.965 (95%CI[0.875–1.064]; *p* = 0.4706). Comparison between the first and last periods continued to show a lowered risk for high-risk patients (RER 0.759 (95%CI[0.598–0.963]); *p* = 0.0266).

Only age remained a significant factor in intermediate (RER 1.314 (95%CI[1.075–1.608]); *p* = 0.0078) and high-risk non-ccRCC (RER 1.134 (95%CI[1.011–1.273]); *p* = 0.0313). In mRCC, male sex (RER 0.924 (95%CI[0.885–0.965]); *p* = 0.0004) was a significant factor for improved prognosis. Whereas older age (RER 1.250 (95%CI[1.199–1.304]); *p* < 0.0001) and period of diagnosis (2006–2014 RER 1.184 (95%CI[1.130–1.241]); *p* < 0.0001, 2015–2019 RER 1.222 (95%CI[1.149–1.300]); *p* < 0.0001) were associated with a lowered prognosis.

## 4. Discussion

Significant changes in RCC diagnosis and treatment have been implemented during the last two decades. The impact of these changes should be evaluated on a large population of affected patients, encompassing the full range of clinical stages and histology groups. The results of this study, based on more than 170,000 cases, show that 5-year RS improved from 79.6% in 2000–2005 to 85.1% in 2015–2019 (all RCC). This sums up to 11,632 life-years gained (incidence ≈ 12,000 cases per year, assuming a life expectancy of another 17.64 years at a mean age of 68 years at diagnosis). These results correspond to those found in other large-scale epidemiological studies and reports [6,23,24]. Healthcare access in Germany can be considered representative for other Western countries, and studies from Scandinavian countries reported similar findings [9,25,26]. Especially striking are the similarities to the results of the DaRenCa study from Denmark, indicating that these findings are common to RCC patients from healthcare systems with equivalent access and care [27].

However, there are several shifts in patient demographic and tumor characteristics to consider. Most patients are diagnosed with low-risk RCC up to 7 cm in diameter, restricted to the renal capsule, and the proportion of cases in this low-risk group has increased from 49.2% in 2000–2005 to 55.1% in 2015–2019. These low-risk cases have a high overall impact on the survival of the total RCC patient population. The main treatment in this group consists of surgical tumor removal, and through follow-up surveillance, RS even increases above expected survival. A significant drop is also seen in the proportion of mRCC by 6% (see Table 1). Both factors are likely to have a high impact on overall prognosis.

In contrast, an increasing age at diagnosis from a median of 66 to 68 years coincides with an increase in life expectancy for the German population in the same period. Therefore, this factor is rather unlikely to affect prognosis (Statistisches Bundesamt (Destatis), 2025, https://www-genesis.destatis.de/datenbank/online/statistic/12621/details; accessed March, 2024).

It becomes evident that the observed improvements in prognosis are mainly the result of increased incidental and early detection of localized and metastatic disease stages. For reference, the changes in the unselected age-standardized incidence and mortality (World Segi) of German kidney cancer patients over time are illustrated in Supplementary Figure S2, as well as the crude mortality-to-incidence ratio.

Regarding a shift in histology from non-ccRCC to ccRCC, there are no indications that there are real changes in tumor characteristics, since the distribution among grading categories or the proportion of specific tumor morphologies such as transitional cell or collecting duct remain stable over all three time periods. A similar change in proportions is seen when performing the same analysis on SEER data from the United States, although the changes are less pronounced (ICD-O3 RCC NOS 8312.3: 46.0% 2000–2005, 21.9% 2015–2019). This shift also offers a reasonable explanation for the decreases in mRCC prognosis since a considerable number of patients with a more favorable prognosis of ccRCC were likely classified as non-ccRCC in the period between 2000 and 2005. It is difficult to determine if these changes are based on histological or documentation practices. To our knowledge this study represents the first to quantify these trends in a nationwide dataset according to individual RCC risk groups. Quantifying trends in individual risk groups, especially for metastasized non-ccRCC subgroups, is crucial since these patients are not as frequently represented in clinical trials.

The data on treatment were limited to yes/no responses for surgical or drug treatments (see Supplementary Table S1). Although limiting for the analysis, the available data showed a consistent pattern for surgical treatment, with nearly 90% of all patients receiving surgery. This proportion was stable over all demographic subgroups and time periods. Patients not receiving surgery were slightly older and presented with mRCC disease at diagnosis. A significant trend over time was seen that less surgery was documented for mRCC patients (2000–2005, 80.5%; 2006–2014, 78.0%; 2015–2019, 69.4%; *p* > 0.0001). Data on specific surgical procedures were not available and could not be evaluated.

A descriptive analysis of non-surgical treatment data showed that in only 1.6% of high-risk and 18.1% of mRCC patients diagnosed during 2015–2019 immunotherapy is reported. However, it is unlikely that the new immunotherapy regimens have yet realized their full potential in this cohort of patients. Several effects need to be considered in this regard: a slow implementation after initial approval, a small target group (young aged, low comorbidity load, advanced ccRCC), and high rates of premature treatment discontinuation. This explains in part why the prognosis of the entire RCC patient cohort has not improved as one might have expected based on clinical study data. This finding has been reported elsewhere, although RCC in general is considered a tumor that is highly responsive to immune-based treatment [28].

One group that does appear to profit from drug treatment advances is high-risk ccRCC patients, a group that is the focus of several immunotherapy trials such as the KEYNOTE-564 study, where yet more improvements in OS were observed [13].

Nevertheless, the smaller but stable subgroup of mRCC patients has not profited from these treatment advances and continues to have a similar or even lower prognosis as nearly two decades ago. This may be due to an increasing comorbidity load as suggested by the increasing first-year mortality. Two important factors to consider are obesity and diabetes. In Germany 19% are considered obese (obesity defined as BMI ≥ 30 kg/m^2^), and 11–12% are diagnosed with diabetes, with an increasing comorbidity burden in older individuals [29,30]. Hypertension as a further risk factor seems well-controlled in Germany, according to a publication from 2022 [31].

The retrospective nature of the study design carries inherent limitations. For example, histology classification has become much more precise. In addition, due to the lack of reliable treatment and comorbidity data, we can only estimate the overall impact of these factors on patient survival. Active surveillance as an option for elderly patients is not routinely documented in the database and is frequently part of the “Missing” category. Physicians, researchers, and medical specialty associations should ensure that cancer registry data include this type of intervention in their documentation. Looking to the near future, this study makes a plea for evolving and up-to-date cancer registry documentation and the routine implementation of similar studies at short intervals to use available data to evaluate and improve healthcare.

## Figures and Tables

**Table 1:** Patient demographic and tumor biology by period of diagnosis. Effect sizes determined using Cramer’s V for categorical values and Cohen’s f for numerical values.

		Period of Diagnosis				Total
		2000–2005	2006–2014	2015–2019		
		*N* (%)	*N* (%)	*N* (%)	*p*-value (effect size)	*N* (%)
		41,290 (19.6)	106,644 (50.7)	62,484 (29.7)		210,418
**Sex**	Male	25,991 (63.0)	68,063 (63.8)	41,172 (65.9)	<0.0001 ^a^ (0.0231)	135,226 (64.3)
	Female	15,299 (37.0)	38,581 (36.2)	21,312 (34.1)		75,192 (35.7)
**Age (median, q1–q3)**		66 (59–73)	69 (59–75)	68 (59–76)	<0.0001 ^b^ (0.0051)	68 (59–75)
**Age groups**	≤44	2000 (4.8)	4259 (4.0)	2258 (3.6)	<0.0001 ^c^ (0.0752)	8517 (4.0)
	45–54	5245 (12.7)	12,650 (11.9)	6998 (11.2)		24,893 (11.8)
	55–64	10,998 (26.6)	23,689 (22.2)	15,038 (24.1)		49,725 (23.6)
	65–74	15,098 (36.6)	36,773 (34.5)	18,220 (29.2)		70,091 (33.3)
	>75	7949 (19.2)	29,273 (27.4)	19,970 (32.0)		57,192 (27.2)
**c/pT**	T1	21,585 (61.0)	60,982 (65.2)	37,622 (68.0)	<0.0001 ^c^ (0.0377)	120,189 (65.3)
	T2	4128 (11.7)	8850 (9.5)	5107 (9.2)		18,085 (9.8)
	T3	9045 (25.6)	22,081 (23.6)	11,630 (21.0)		42,756 (23.2)
	T4	621 (1.8)	1556 (1.7)	962 (1.7)		3139 (1.7)
	*Missing*	*5911 (14.3)*	*13,175 (12.3)*	*7163 (11.5)*		*26,249 (12.5)*
**c/pN**	N0	22,016 (91.7)	49,747 (91.0)	33,943 (92.1)	0.0084 ^a^ (0.0173)	105,706 (91.5)
	N+	1992 (8.3)	4895 (9.0)	2901 (7.9)		9788(8.5)
	*NX/Missing*	*17,282 (41.9)*	*52,002 (48.8)*	*25,640 (41.0)*		*94,924 (45.1)*
**c/pM**	M0	19,471 (81.4)	52,420 (83.5)	38,511 (86.1)	<0.0001 ^a^ (0.0465)	110,402 (84.0)
	M1a–c	4458 (18.6)	10,356 (16.5)	6207 (13.9)		21,021 (16.0)
	*Missing*	*17,361 (42.0)*	*43,868 (41.1)*	*17,766 (28.4)*		*78,995 (37.5)*
**Histology**	Clear cell	9879 (23.9)	50,535 (47.4)	35,438 (56.7)	<0.0001 ^c^ (0.2402)	95,852 (45.5)
	Papillary	1202 (2.9)	10,254 (9.6)	8677 (13.9)		20,133 (9.6)
	Chromophobe	882 (2.1)	4678 (4.4)	3662 (5.9)		9222 (4.4)
	NOS/Other	29,129 (70.5)	40,543 (38.0)	14,338 (23.0)		84,010 (39.9)
	Collecting duct	90 (0.2)	370 (0.3)	183 (0.3)		643 (0.3)
	Transitional cell	106 (0.3)	260 (0.2)	174 (0.3)		540 (0.3)
	Molecular defined	2 (0.0)	4 (0.0)	12 (0.0)		18 (0.0)
**Grade**	G1	7452 (20.7)	18,674 (19.6)	12,302 (23.3)	<0.0001 ^c^ (0.0480)	38,428 (20.9)
	G2	23,152 (64.2)	60,119 (63.2)	30,890 (58.5)		114,161 (62.0)
	G3	5211 (14.4)	15,200 (16.0)	8430 (16.0)		28,841 (15.7)
	Anaplastic	247 (0.7)	1118 (1.2)	1177 (2.2)		2542 (1.4)
	*Missing*	*5228 (12.7)*	*11,533 (10.8)*	*9685 (15.5)*		*26,446 (12.6)*
**UISS-risk**	Low	11,569 (49.2)	32,269 (52.7)	22,531 (55.1)	<0.0001 ^c^ (0.0315)	66,369 (52.8)
	Intermediate	3103 (13.2)	7559 (12.3)	5544 (13.5)		16,206 (12.9)
	High	3344 (14.2)	8704 (14.2)	5468 (13.4)		17,516 (13.9)
	mRCC	5482 (23.3)	12,709 (20.7)	7377 (18.0)		25,568 (20.3)
	*Missing*	*17,792 (43.1)*	*45,403 (42.6)*	*21,564 (34.5)*		*84,759 (40.3)*

^a^, Cochran–Armitage test; ^b^, ANOVA; ^c^, χ2-test.

**Table 2:** Regression model of excess mortality for each UISS-risk category adjusted for patient characteristics and follow-up intervals.

	UISS-Risk Category									mRCC		
	Low			Intermediate			High					
Factor	RER	95% CI	*p*-Value	RER	95% CI	*p*-Value	RER	95% CI	*p*-Value	RER	95% CI	*p*-Value
**Sex**			0.6577			0.8019			0.0430			0.0008
Female	ref	-		ref	-		ref	-		ref	-	
Male	1.077	0.775–1.498		1.021	0.868–1.200		0.909	0.829–0.997		0.942	0.909–0.975	
**Age at** **diagnosis**			0.0739			0.0018			0.0286			<0.0001
<65	ref	-		ref	-		ref	-		ref	-	
≥65	0.663	0.422–1.040		1.289	1.099–1.511		1.106	1.010–1.211		1.224	1.183–1.266	
**Period of** **diagnosis**			0.0549			0.1772			0.0009			<0.0001
2000–2005	ref	-		ref	-		ref	-		ref	-	
2006–2014	0.934	0.647–1.341	0.7021	0.895	0.745–1.075	0.2357	0.933	0.841–1.035	0.1886	1.099	1.055–1.145	<0.0001
2015–2019	0.482	0.229–1.015	0.0549	0.796	0.624–1.016	0.0668	0.771	0.669–0.888	0.0003	1.035	0.985–1.088	0.1694

CI, 95% confidence interval; mRCC, metastatic renal cell carcinoma; ref., reference category; RER, relative excess risk.

## Data Availability

A.S.R. and K.H. had full access to all the data in this study and took responsibility for the integrity of these data and the accuracy of the data analysis. Data are not available to other researchers. The data are part of the RKI Cancer Registry Database that is routinely collected and can be accessed through an application process.

## Supplementary Materials

The following supporting information can be downloaded at: https://www.mdpi.com/article/10.3390/curroncol32090515/s1, Figure S1: Patient flowchart. Exclusion criteria were applied according to the order illustrated in the plot. The absolute DCO-rate was 10.7% (N = 27,089); Table S1: Baseline RCC cohort. Overview of documented treatment according to demographic and disease characteristics; Figure S2: Unselected ICD 64 overall age-standardized (World Segi) incidence and mortality rates for Germany as well as the crude mortality-to-incidence ratio of the corresponding case numbers on the secondary y-axis.

## Abbreviations

ccRCC	clear cell renal carcinoma
DCO	death certificate only
IQ-range	interquartile range (Q_1_–Q_3_)
mRCC	metastatic renal cell carcinoma
NET	neuroendocrine tumor
Non-ccRCC	non-clear cell renal carcinoma
RCC	renal cell carcinoma
RER	relative excess risk
TKI	tyrosine kinase inhibitor
UISS	University of California Los Angeles integrated staging system
