# Supplementary material for: Renal Cell Carcinoma: Prognosis in the Era of Targeted Therapy
Source: Curr Oncol. 2025 Sep 16;32(9):515. doi: 10.3390/curroncol32090515 (PMC12468357; doi:10.3390/curroncol32090515)
Supplement: Supplementary file 1 [file curroncol-32-00515-s001.zip › curroncol-3865302-supplementary.pdf]

Supplementary Material

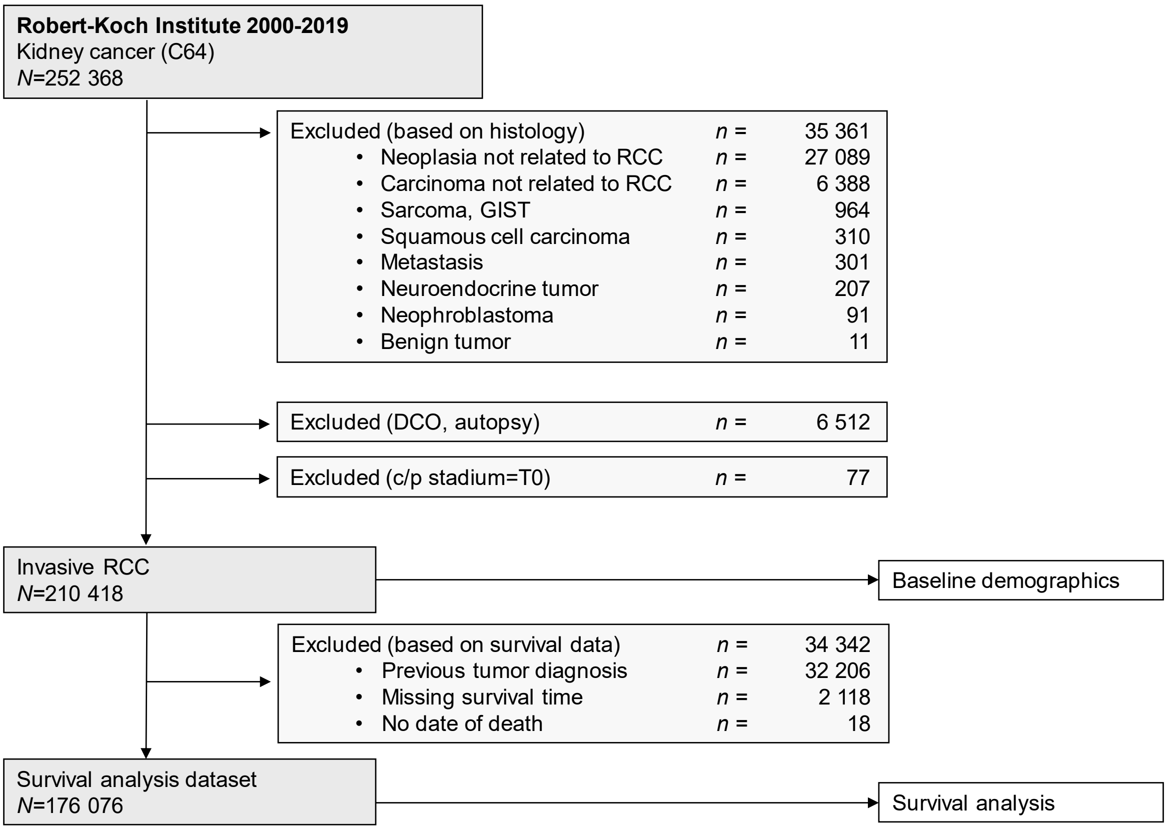

Figure S1: *Patient flowchart. Exclusion criteria were applied according to the order illustrated in the plot. The absolute DCO-rate was 10.7% (N=27,089).*

Table S1: *Baseline RCC cohort. Overview of documented treatment according to demographic and disease characteristics.*

		Surgery ***N* (%)**			Immunotherapy ***N* (%)**			Other ***N* (%)**	
	Yes	No	*Missing*	Yes	No	*Missing*	Yes	No	*Missing*
**Total**	115 247 (90.4)	12 281 (11.9)	*82 890 (39.4)*	1 904 (2.1)	89 048 (97.9)	*119 466 (56.8)*	7 411 (16.6)	37 328 (83.4)	*165 679 (78.7)*
**Sex**									
Male	73 926 (90.4)	7 887 (9.6)	*53 413 (39.5)*	1 300 (2.2)	56 627 (97.7)	*77 299 (57.2)*	4 812 (16.6)	24 211 (83.4)	*106 203 (78.5)*
Female	41 321 (90.4)	4 394 (9.6)	*29 477 (39.2)*	604 (1.8)	32 421 (98.2)	*42 167 (56.1)*	2 599 (16.5)	13 117 (83.5)	*59 476 (79.1)*
**Age (median, [IQ-range])**	67 (59-75)	70 (62-77)	*68 (59-76)*	65 (57-73)	68 (59-75)	*68 (59-75)*	67 (59-74)	68 (59-75)	*68 (59-75)*
**UISS-risk category**									
Low	47 542 (97.1)	1 400 (2.9)	*17 427 (26.3)*	45 (0.1)	35 201 (99.9)	*31 123 (46.9)*	1 758 (12.6)	12 134 (87.3)	*52 477 (79.1)*
Intermediate	11 371 (96.9)	362 (3.1)	*4 473 (27.6)*	51 (0.6)	8 421 (99.4)	*7 734 (47.7)*	496 (14.4)	2 955 (85.6)	*12 755 (78.7)*
High	12 566 (97.0)	382 (3.0)	*4 568 (26.1)*	134 (1.5)	9 010 (98.5)	*8 372 (47.8)*	704 (17.7)	3 273 (82.3)	*13 539 (77.3)*
mRCC	13 176 (78.6)	3 584 (21.4)	*8 808 (34.4)*	1 500 (11.7)	11 304 (88.3)	*12 764 (49.9)*	2 778 (40.0)	4 164 (60.0)	*18 626 (72.8)*
Missing	*30 592 (82.4)*	*6 553 (17.6)*	*47 614 (56.2)*	*174 (0.7)*	*25 122 (99.3)*	*59 473 (70.2)*	*1 675 (10.2)*	*14 802 (89.8)*	*68 282 (80.6)*
**Period of diagnosis**									
2000-2005	24 336 (90.2)	2 643 (9.8)	*14 311 (34.7)*	320 (1.5)	20 477 (98.5)	*20 493 (49.6)*	2 507 (28.8)	6 187 (71.2)	*32 596 (78.9)*
2006-2014	60 993 (91.6)	5 604 (8.4)	*40 047 (37.5)*	774 (1.8)	42 914 (98.2)	*62 956 (59.0)*	3 855 (16.0)	20 271 (84.0)	*82 518 (77.4)*
2015-2019	29 918 (88.1)	4 034 (11.9)	*28 532 (45.7)*	810 (3.1)	25 657 (96.9)	*36 017 (57.6)*	1 049 (8.8)	10 870 (91.2)	*50 565 (80.9)*

IQ, Interquartile range, mRCC, Metastatic renal cell carcinoma, UISS, UCLA integrated staging system

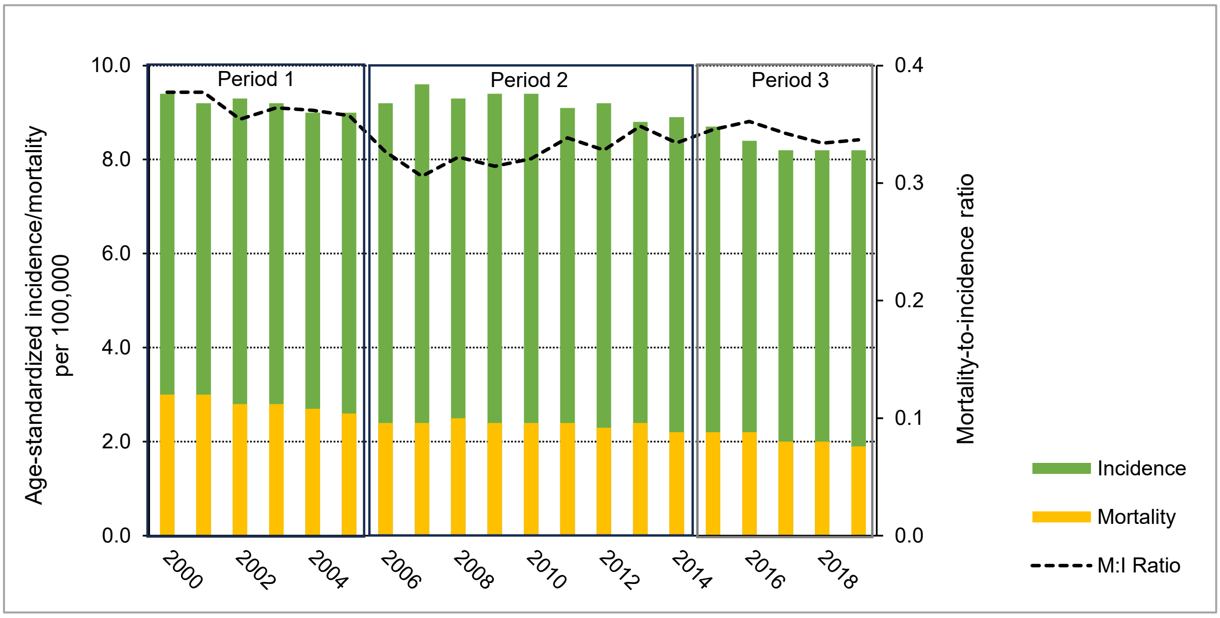

Figure S2: *Unselected ICD 64 overall age-standardized (World Segi) incidence and mortality rates for Germany as well as the crude mortality-to-incidence ratio of the corresponding case numbers on the secondary y-axis. Data extracted in February 2025 (https://www.krebsdaten.de/Krebs/EN/Database/databasequery_step1_node.html).*
